# Quality of life of COVID-19 recovered patients: a 1-year follow-up study from Bangladesh

**DOI:** 10.1186/s40249-023-01125-9

**Published:** 2023-08-25

**Authors:** Mohammad Delwer Hossain Hawlader, Md Utba Rashid, Md Abdullah Saeed Khan, Mowshomi Mannan Liza, Sharmin Akter, Mohammad Ali Hossain, Tajrin Rahman, Sabrina Yesmin Barsha, Alberi Afifa Shifat, Mosharop Hossian, Tahmina Zerin Mishu, Soumik Kha Sagar, Ridwana Maher Manna, Nawshin Ahmed, Sree Shib Shankar Devnath Debu, Irin Chowdhury, Samanta Sabed, Mashrur Ahmed, Sabrina Afroz Borsha, Faraz Al Zafar, Sabiha Hyder, Abdullah Enam, Habiba Babul, Naima Nur, Miah Md. Akiful Haque, Shopnil Roy, K. M. Tanvir Hassan, Mohammad Lutfor Rahman, Mohammad Hayatun Nabi, Koustuv Dalal

**Affiliations:** 1 Department of Public Health, North South University, Bashundhara, Dhaka, 1229 Bangladesh; 2 International Centre for Diarrhoeal Disease Research, Bangladesh, Mohakhali, Dhaka, 1212 Bangladesh; 3 National Institute of Preventive and Social Medicine, Mohakhali, Dhaka, 1212 Bangladesh; 4 Ibn Sina Medical College Hospital, Kallyanpur, Dhaka, 1216 Bangladesh; 5 Public Health Promotion and Development Society (PPDS), Dhaka, 1205 Bangladesh; 6 Covid Vaccine Coordination Cell, Directorate General of Health Services (DGHS), Dhaka, Bangladesh; 7 Institute of Statistical Research and Training (ISRT), University of Dhaka, Dhaka, 1000 Bangladesh; 8 School of Health Sciences, Division of Public Health Science, Mid Sweden University, Sundsvall, Sweden

**Keywords:** Quality of life, Health-related quality of life, Reverse transcription-polymerase chain reaction, COVID-19, Bangladesh

## Abstract

**Background:**

The COVID-19 pandemic posed a danger to global public health because of the unprecedented physical, mental, social, and environmental impact affecting quality of life (QoL). The study aimed to find the changes in QoL among COVID-19 recovered individuals and explore the determinants of change more than 1 year after recovery in low-resource settings.

**Methods:**

COVID-19 patients from all eight divisions of Bangladesh who were confirmed positive by reverse transcription-polymerase chain reaction from June 2020 to November 2020 and who subsequently recovered were followed up twice, once immediately after recovery and again 1 year after the first follow-up. The follow-up study was conducted from November 2021 to January 2022 among 2438 individuals using the World Health Organization Quality of Life Brief Version (WHOQOL-BREF). After excluding 48 deaths, 95 were rejected to participate, 618 were inaccessible, and there were 45 cases of incomplete data. Descriptive statistics, paired-sample analyses, generalized estimating equation (GEE) analysis, and multivariable logistic regression analyses were performed to test the mean difference in participants’ QoL scores between the two interviews.

**Results:**

Most participants (*n* = 1710, 70.1%) were male, and one-fourth (24.4%) were older than 46. The average physical domain score decreased significantly from baseline to follow-up, and the average scores in psychological, social, and environmental domains increased significantly at follow-up (*P* < 0.05). By the GEE equation approach, after adjusting for other factors, we found that older age groups (*P* < 0.001), being female (*P* < 0.001), having hospital admission during COVID-19 illness (*P* < 0.001), and having three or more chronic diseases (*P* < 0.001), were significantly associated with lower physical and psychological QoL scores. Higher age and female sex [adjusted odd ratio (a*OR*) = 1.3, 95% confidence interval (*CI*) 1.0–1.6] were associated with reduced social domain scores on multivariable logistic regression analysis. Urban or semi-urban people were 49% less likely (a*OR* = 0.5, 95% *CI* 0.4–0.7) and 32% less likely (a*OR* = 0.7, 95% *CI* 0.5–0.9) to have a reduced QoL score in the psychological domain and the social domain respectively, than rural people. Higher-income people were more likely to experience a decrease in QoL scores in physical, psychological, social, and environmental domains. Married people were 1.8 times more likely (a*OR* = 1.8, 95% *CI* 1.3–2.4) to have a decreased social QoL score. In the second interview, people admitted to hospitals during their COVID-19 infection showed a 1.3 times higher chance (a*OR* = 1.3, 95% *CI* 1.1–1.6) of a decreased environmental QoL score. Almost 13% of participants developed one or more chronic diseases between the first and second interviews. Moreover, 7.9% suffered from reinfection by COVID-19 during this 1-year time.

**Conclusions:**

The present study found that the QoL of COVID-19 recovered people improved 1 year after recovery, particularly in psychological, social, and environmental domains. However, age, sex, the severity of COVID-19, smoking habits, and comorbidities were significantly negatively associated with QoL. Events of reinfection and the emergence of chronic disease were independent determinants of the decline in QoL scores in psychological, social, and physical domains, respectively. Strong policies to prevent and minimize smoking must be implemented in Bangladesh, and we must monitor and manage chronic diseases in people who have recovered from COVID-19.

**Graphical Abstract:**

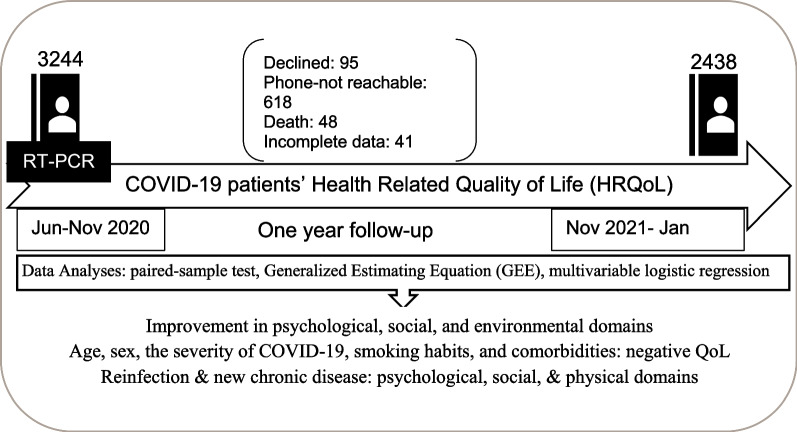

**Supplementary Information:**

The online version contains supplementary material available at 10.1186/s40249-023-01125-9.

## Background

COVID-19 caused by severe acute respiratory syndrome coronavirus 2 (SARS-CoV-2) has been a burden for global health systems, and it has led to personal and social consequences from its onset [1]. Citizens had to follow specific social distancing measures, undergo lockdown scenarios, avoid social gatherings, refrain from crowds or congregations of people, and generally endure restricted living and quality of life [2]. Furthermore, the COVID-19 pandemic posed a danger to global public health because of unprecedented panic, stress, worry, and dissatisfaction concerning health-related quality of life (HRQoL) [3].

In the last decade, QoL has been explored mainly in research specializing in non-communicable and chronic diseases. HRQoL is dynamic, subjective, and multi-dimensional. These dimensions include physical, social, psychological, and environmental considerations [4, 5]. The World Health Organization (WHO) has conceptualized HRQoL as an individual’s belief in their fitness and fitness-associated domains [6]. For instance, chronic diseases with COVID-19 infection may reduce the HRQoL compared to pre-COVID [7]. The WHO noted that many individuals experience persistent symptoms following COVID-19 infection [8]. According to studies conducted in Wuhan, China, about half of the hospitalized patients exhibited nonspecific symptoms, with respiratory difficulties being the most frequent 3 months after discharge [9]. Six months after the onset of symptoms, nearly two-thirds of patients reported fatigue, and one-quarter reported sleeping disturbance [10]. Regardless of the degree of the condition, one-quarter of these individuals had exercise capacity below the usual cutoff point. This scenario replicates previous SARS-CoV infections, characterized by impaired exercise ability and lower QoL as long as 2 years after the initial condition [11, 12].

QoL is a strong predictor of endurance in general health and well-being [13]. Therefore, evaluation of the QoL throughout numerous domains may allow us to identify a variety of issues that could impact an individual’s well-being [14, 15]. A previous report from Hong Kong, China assessed the HRQoL among survivors of SARS 6 months after the pandemic and mentioned significant impairment in HRQoL. Additionally, an observation from Morocco noted poor outcomes in the COVID-19 pandemic on HRQoL [16]. There has been much research since the beginning of COVID-19, but a few studies explored the long-term changes in the QoL of COVID-19 patients’ years after recovery. The study aimed to find the differences in QoL among COVID-19 recovered individuals and examine the determinants of change more than 1 year after recovery.

## Methods

### Study design and study participants

This follow-up study focuses on COVID-19 patients confirmed by reverse transcription-polymerase chain reaction (RT-PCR) from June 2020 to November 2020 and who subsequently recovered. A baseline cross-sectional assessment of QoL using the WHOQOL-BREF [17] instrument was carried out between November 2020 and January 2021 among 3244 COVID-19 recovered participants from eight administrative divisions of Bangladesh. A detailed methodology of the study was described elsewhere [14]. We targeted all the respondents who participated in the baseline survey for the follow-up study. Participants excluded were: (1) those who died before the follow-up visit; (2) those who declined participation; and (3) those who could not be reached (due to a call drop, call waiting, an inactive number, or a network problem). A total of 48 participants had died, 95 individuals rejected participation, and 618 people were inaccessible during the follow-up period. Finally, the data collection team interviewed 2479 people (Additional file 1: Fig. S1). This follow-up survey was administered from mid-November 2021 to late January 2022 (1 year after the baseline survey).

### Data collection procedure

Data were collected using the structured questionnaire prepared during the baseline interview (1st interview), with some modifications. Once the revised questionnaire was finalized, the data collection team was given a list of division enrollees. The 20-person study team conducted over-the-phone interviews with the participants. We assigned interviewers to each division based on location to overcome linguistic obstacles. Before initiating the interviews, we assured the interviewees that questions could be skipped if they felt uncomfortable answering. The quality assurance team was assigned to ensure data accuracy, regular data monitoring, adherence to protocols, and overall research integrity.

### Study instrument

The pre-tested structured questionnaire used during the first interview was slightly modified to include two questions about the vaccination against COVID-19 and the incidence of reinfection between the first and second interviews. The final questionnaire consisted of a socio-demographic profile, personal history, presence of comorbidities, COVID-19 vaccination, and reinfection history. We used the WHOQOL-BREF scale for quality-of-life assessment.

### WHOQOL-BREF

To assess the QoL of COVID-19-positive patients, we used a Bangla-validated version WHOQOL-BREF quality of life assessment questionnaire [18]. The WHOQOL-BREF is the most widely used, cross-culturally sensitive QoL assessment tool that illustrates the individual’s quality of life from participants’ physical, psychological, social, and environmental perspectives 26 items. It was assumed that a higher field score indicated a higher quality of life, so all scores were recorded positively.

### Statistical analysis

The WHOQOL-BREF scores were converted to a scale of 100 based on the guideline (18). Descriptive statistics were expressed as frequency (percentage) or mean (± standard deviation). In analytic statistics, paired-sample analyses of scores between the first and second interviews were conducted to assess the changes in QoL scores over 1 year using a paired-sample *t*-test. In addition, an independent sample *t*-test or Analysis of Variance (ANOVA) was carried out for each interview point to compare QoL scores across the categories of factor variables. A generalized estimating equation (GEE) analysis was carried out to reveal differences in QoL scores across categories of independent variables after adjustment of intra-individual variation between two interview points. The family, link function, and correlation structure for GEE were set to normal, identity, and exchangeable, respectively. We determined whether the score increased, decreased, or remained unchanged for individual patients in the four domains. Determinants of decline in QoL scores from the first to second interview (in four domains separately) were explored through multivariable logistic regression analyses. All statistical tests were carried out in the statistical software Stata version 16 (StataCorp, College Station, TX, USA). Statistical software R Studio (version 2022.07.1) (Lucent Technologies, Jasmine Mountain, USA) and Microsoft Excel Version 2019 (Microsoft Corporation, 1 Microsoft Way, Redmond, WA) were used for creating graphs.

## Results

### Sociodemographic and clinical profile of the participants

**Fig. 1:**
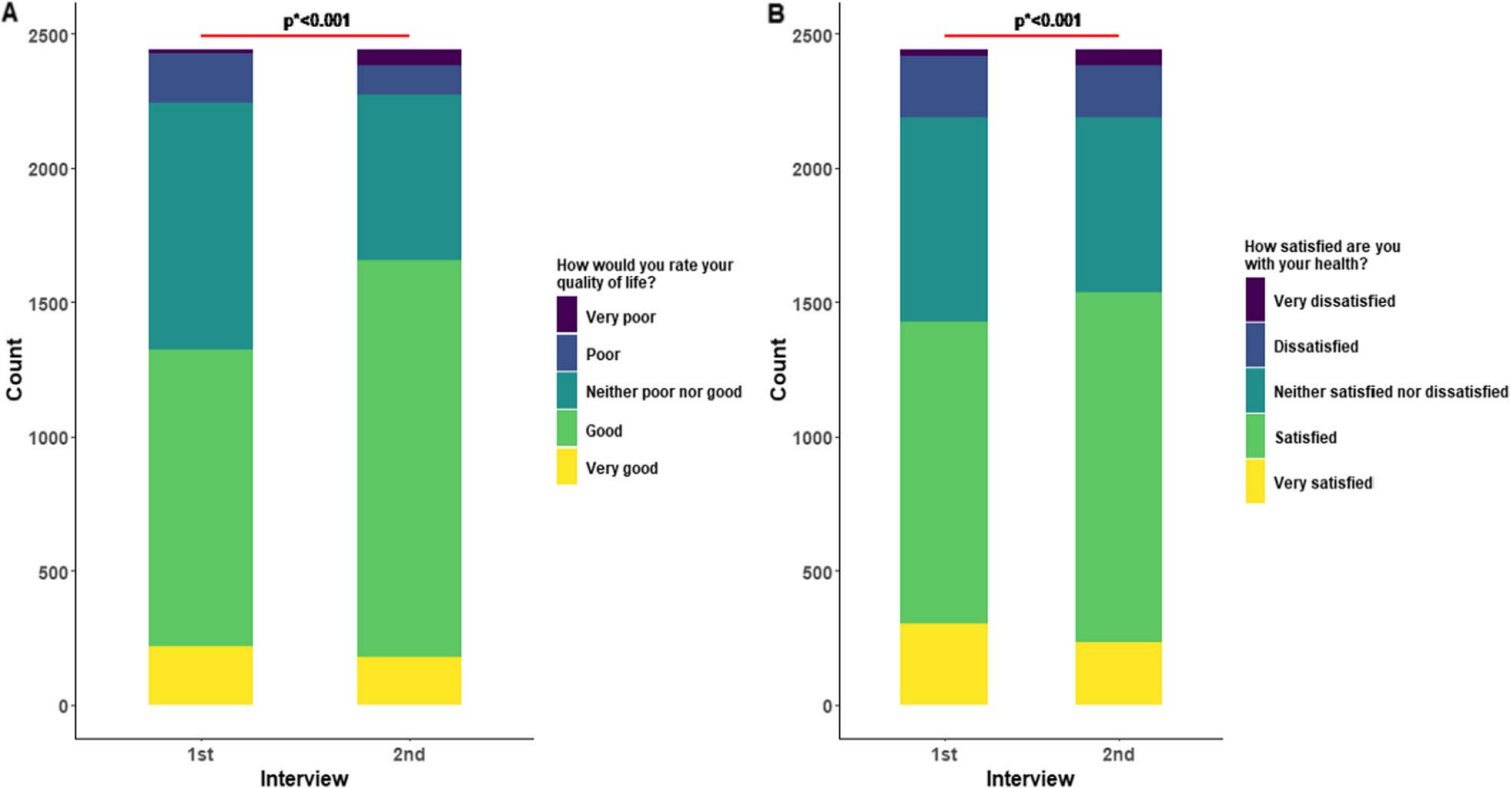
Pattern of changes in overall quality of life and health satisfaction over the study period

Table 1 illustrates our study participants’ demographic and clinical characteristics (*n* = 2438). The average age of the participants at inclusion was 38.1 ± 2.3 years, and the majority were aged more than 46 years (24.4%), male (70.1%), and living in the urban areas (74.5%) of the country. We observed a significant improvement in participants’ QoL in every domain as well as individuals’ overall perception of QoL and their health (as assessed by Q1 and Q2) except the physical domain (Fig. 1). The average physical domain score decreased significantly from baseline to follow-up, whereas the mean scores in psychological, social, and environmental domains increased significantly at follow-up (*P* < 0.05) (Additional file 1: Fig. S2).

**Table 1:** Demographic characteristics and participant clinical profiles

Characteristics	Category	Frequency (*n*)	Percentage (%)
Age	< 26	317	13.0
	26–30	499	20.5
	31–35	418	17.1
	36–40	367	15.0
	41–45	242	9.9
	46+	595	24.4
Gender	Male	1710	70.1
	Female	728	29.9
Division	Barishal	98	4.0
	Chattogram	340	13.9
	Dhaka	1217	49.9
	Khulna	149	6.1
	Mymensingh	129	5.3
	Rajshahi	216	8.9
	Rangpur	146	6.0
	Sylhet	143	5.9
Residence	Rural	315	12.9
	Urban	1816	74.5
	Semi-urban	307	12.6
Educational status	No formal education	57	2.3
	Primary	171	7.0
	Up to SSC	270	11.1
	Up to HSC	598	24.5
	Graduation	920	37.7
	Post-graduation	422	17.3
Employment status	Service	1417	58.1
	Business	350	14.4
	Farmer	27	1.1
	Housewife	307	12.6
	Student	173	7.1
	Unemployed	88	3.6
	Others	76	3.1
Monthly family income in BDT (*1 USD* = *105 BDT*)	≤ 20 000	595	24.4
	20 001–40 000	1012	41.5
	40 001–60 000	450	18.5
	> 60 000	381	15.6
Marital status	Single	438	18.0
	Married	1945	79.8
	Separated	4	0.2
	Divorced	18	0.7
	Widowed/widower	33	1.3
Health care worker	No	2067	84.8
	Yes	371	15.2
Smoke	No	1616	66.3
	Yes	533	21.9
	Past smoker	289	11.8
Hypertension	No	2000	82.0
	Yes	438	18.0
Diabetes mellitus	No	2045	83.9
	Yes	393	16.1
Heart diseases	No	2263	92.8
	Yes	175	7.2
Asthma	No	2173	89.1
	Yes	265	10.9
Chronic kidney disease	No	2360	96.8
	Yes	78	3.2
Cancer	No	2208	96.5
	Yes	81	3.5

*BDT* Bangladesh Taka, *USD* United States Dollar

### Quality of life dynamics of participants

Table 2 describes the inter and intra-interview change of the participants’ QoL across different variables over the period. The physical domain scores decreased significantly among those aged ≥ 36 years, educated, employed, and married, irrespective of sex, living area, and hospital admission. The psychological domain score increased significantly at follow-up in participants aged < 46 years (*P* < 0.001), living in urban/semi-urban areas (*P* < 0.001), participants having graduation (or above) (*P* < 0.002), single (not-married) individuals (*P* < 0.02), health care workers (HCWs) (*P* < 0.001), and persons having a history of hospital admission (*P* < 0.001). The social domain scores improved significantly in participants aged < 36 years (*P* < 0.001), irrespective of sex (*P* < 0.001), living area (*P* < 0.001), marital status (*P* < 0.001), and smoking history (*P* < 0.001), in individuals with education (*P* < 0.001), among employed participants (*P* < 0.001), among those having a monthly family income of < 60 000 Bangladesh Taka (BDT) (*P* < 0.001), and among those without a history of hospital admission (*P* < 0.001). Participants with a history of hospital admission showed significant declines (*P* < 0.001) in social domain scores. For the environmental domain, the average scores increased significantly (*P* < 0.001) for all the variables except the participants who were uneducated, unemployed, living in rural areas, and had a previous smoking history.

**Table 2:** Comparison of quality of life between baseline and follow-up interviews

Variable		Physical			Psychological			Social			Environmental		
		1st visit	2nd visit	*P*	1st visit	2nd visit	*P*	1st visit	2nd visit	*P*	1st visit	2nd visit	*P*
		Mean (*SD*)	Mean (*SD*)		Mean (*SD*)	Mean (*SD*)		Mean (*SD*)	Mean (*SD*)		Mean (*SD*)	Mean (*SD*)	
Overall domain score		69.2 (14.2)	66.7 (14.5)	< 0.001	64.3 (15.3)	65.1 (14.0)	0.02	61.5 (19.4)	65.8 (17.5)	< 0.001	62.9 (12.8)	65.9 (11.5)	< 0.001
Age	< 26	71.7 (14.2)	71.9 (14.1)	0.80	66.1 (15.6)	69.6 (13.9)	0.001	44.2 (18.1)	53.4 (17.5)	< 0.001	63.1 (13.6)	66.4 (11.0)	< 0.001
	26–35	70.5 (14.0)	69.5(13.6)^a^	0.07	65.9 (14.9)	67.1 (13.5)^a^	0.04	61.5 (20.0)^a^	68.6 (17.7)^a^	< 0.001	63.1 (12.8)	66.3 (11.4)	< 0.001
	36–45	70.6(13.4)	65.5 (14.7)^ab^	< 0.001	64.4 (15.1)	64.0 (14.5)^ab^	0.59	68.8 (15.9)^ab^	69.3 (15.3)^a^	0.54	63.4 (12.1)	65.9 (12.4)	< 0.001
	≥ 46	64.2 (14.0)^abc^	60.8 (13.9)^abc^	< 0.001	60.6 (15.4)^abc^	60.8 (13.3)^abc^	0.80	63.5 (16.6)^ac^	64.3 (16.2)^abc^	0.33	62.1 (13.0)	64.9 (10.9)	< 0.001
	*P-value*	< 0.001	< 0.001		< 0.001	< 0.001		< 0.001	< 0.001		0.38	0.15	
Gender	Male	69.7 (14.3)	67.5 (14.4)	< 0.001	65.2 (15.4)	66.0 (13.9)	0.06	60.9 (19.3)	66.0 (17.4)	< 0.001	62.9 (12.8)	65.9 (11.3)	< 0.001
	Female	68.0 (13.9)	64.8 (14.7)	< 0.001	62.1 (15.1)	62.9 (14.2)	0.18	62.8 (19.7)	65.1 (17.7)	0.01	62.8 (12.7)	65.8 (11.9)	< 0.001
	*P-value*	0.01	< 0.001		< 0.001	< 0.001		0.02	0.26		0.79	0.84	
Residence	Rural	71.5 (13.5)	67.5 (15.7)	< 0.001	66.9 (13.4)	62.8 (15.6)	< 0.001	61.5 (17.6)	64.5 (16.9)	0.01	61.9 (12.6)	63.2 (10.9)	0.17
	Urban/semi-urban	68.8 (14.3)	66.6 (14.4)	< 0.001	63.9 (15.6)	65.5 (13.8)	< 0.001	61.5 (19.7)	65.9 (17.6)	< 0.001	63.1 (12.8)	66.3 (11.5)	< 0.001
	*P-value*	0.002	0.28		0.001	0.002		0.99	0.16		0.12	< 0.001	
Education	No or primary education	67.5 (14.9)	65.7 (16.2)	0.19	61.6 (15.4)	59.5 (13.6)	0.08	60.4 (17.6)	60.8 (17.3)	0.78	62.0 (14.9)	61.8 (11.6)	0.88
	Up to HSC	68.6 (14.2)	66.3 (14.7)	< 0.001	63.8 (15.0)	64.5 (14.6)^a^	0.28	58.5 (19.7)	63.5 (17.3)	< 0.001	61.9 (12.22)	64.7 (10.9)^a^	< 0.001
	Graduate/above	69.8 (14.0)	67.1 (14.1)	< 0.001	65.0 (15.5)^a^	66.5 (13.5)^ab^	0.002	63.7 (19.1)^b^	68.1 (17.3)^ab^	< 0.001	63.8 (12.7)^b^	67.3 (11.6)^ab^	< 0.001
	*P-value*	0.02	0.24		0.01	< 0.001		< 0.001	< 0.001		0.002	< 0.001	
Employment status	Unemployed	65.7 (14.9)	64.6 (16.5)	0.62	60.8 (15.0)	61.9 (14.2)	0.59	57.9 (19.5)	59.8 (19.4)	0.44	62.7 (12.2)	64.7 (10.6)	0.29
	Employed	69.3 (14.1)	66.8 (14.5)	< 0.001	64.4 (15.3)	65.2 (14.0)	0.02	61.7 (19.4)	65.9 (17.4)	< 0.001	62.9 (12.8)	65.9 (11.5)	< 0.001
	*P-value*	0.02	0.18		0.03	0.03		0.08	0.001		0.87	0.33	
Monthly family income (BDT)	≤ 20 000	67.3 (14.8)	65.9 (14.6)	0.06	61.4 (15.5)	64.6 (13.7)	< 0.001	55.9 (21.0)	64.2 (17.4)	< 0.001	61.9 (14.9)	63.9 (10.2)	0.01
	20 001–40 000	69.4 (13.7)^a^	67.7 (14.6)	0.004	65.0 (14.2)^a^	65.7 (13.9)	0.20	61.9 (18.0)^a^	66.0 (17.4)	< 0.001	61.6 (12.0)	65.6 (11.4)^a^	< 0.001
	40 001–60 000	70.3 (14.0)^a^	67.5 (14.5)	< 0.001	66.7 (16.0)^a^	66.0 (14.1)	0.44	62.6 (19.1)^a^	66.6 (17.7)	< 0.001	64.6 (12.1)^b^	67.6 (11.6)^ab^	< 0.001
	> 60 000	70.2 (14.4)^a^	64.1 (13.9)^bc^	< 0.001	63.8 (16.5)^c^	63.1 (14.7)^bc^	0.46	67.9 (18.4)^abc^	66.5 (17.6)	0.23	66.1 (11.1)^ab^	67.7 (13.0)^ab^	0.07
	*P-value*	0.002	< 0.001		< 0.001	0.01		< 0.001	0.08		< 0.001	< 0.001	
Marital status	Single	70.9 (14.9)	70.2 (15.0)	0.33	65. (15.9)	67.7 (14.6)	0.02	38.3 (12.9)	51.4 (18.7)	< 0.001	63.3 (13.1)	66.8 (11.6)	< 0.001
	Married	68.7 (13.9)	65.8 (14.3)	< 0.001	63.9 (15.1)	64.5 (13.8)	0.15	67.4 (16.1)	69.4 (15.1)	< 0.001	62.8 (12.7)	65.6 (11.5)	< 0.001
	*P-value*	0.002	< 0.001		0.01	< 0.001		< 0.001	< 0.001		0.45	0.04	
Healthcare workers	No	69.2 (14.4)	66.3 (14.5)	< 0.001	64.5 (15.5)	64.5 (14.1)	0.95	61.5 (19.2)	65.2 (17.4)	< 0.001	63.0 (13.0)	65.5 (11.5)	< 0.001
	Yes	68.9 (13.3)	68.5 (14.5)	0.65	62.5 (14.0)	68.3 (13.3)	< 0.001	61.7 (20.4)	68.9 (17.5)	< 0.001	62.5 (11.6)	67.7 (11.6)	< 0.001
	*P-value*	0.75	0.01		0.02	< 0.001		0.884	< 0.001		0.51	< 0.001	
Hospital admission	No	70.8 (14.0)	68.3(14.0)	< 0.001	66.1 (14.8)	66.2 (13.9)	0.93	62.9 (19.5)	66.5 (17.5)	< 0.001	62.8 (12.2)	66.3 (11.5)	< 0.001
	Yes	65.7 (13.9)	63.2 (15.0)	< 0.001	60.3 (15.7)	62.9 (14.0)	< 0.001	68.5 (18.8)	64.3 (17.3)	< 0.001	63.1 (13.9)	65.0 (11.5)	0.003
	*P-value*	< 0.001	< 0.001		< 0.001	< 0.001		< 0.001	0.003		0.60	0.01	
Smoking status	No	69.1 (14.)	66.5 (14.3)	< 0.001	64.2 (14.8)	64.9 (13.9)	0.11	62.2 (19.5)	65.7 (17.6)	< 0.001	62.8 (12.6)	65.9 (11.2)	< 0.001
	Yes	68.32 (14.4)	66.5 (14.8)	0.02	61.9 (15.9)^a^	65.3 (13.6)	< 0.001	58.7 (19.4)^a^	64.9 (17.4)	< 0.001	62.2 (13.6)	65.5 (11.9)	< 0.001
	Past smoker	71.0 (14.4)^b^	68.1 (15.4)	0.01	68.8 (16.1)^ab^	65.9 (15.4)	0.02	63.1 (18.2)^b^	67.7 (16.8)	< 0.001	64.9 (12.5)^ab^	66.0 (12.4)	0.26
	*P-value*	0.03	0.23		< 0.001	0.48		< 0.001	0.08		0.01	0.73	

Scores were expressed as mean ± *SD*

*SD* Standard deviation, *BDT* Bangladesh Taka, *HCW* Healthcare worker

*P* value was determined using one-way ANOVA with post-hoc analysis by Duncan multiple range test

^a^^−^^c^Scores with different superscript letters have a statistically significant difference across categories of the variable within a domain, e.g., values with a superscript ‘a’ is significantly different from values with other superscript(s)

### Quality of life dynamics of participants with chronic disease

Of all, 13.1% of participants developed one or more chronic diseases between the first and second interviews, and 7.9% were re-infected from COVID-19 during the follow-up period (Additional file 1: Fig. S3). In the physical domain, the participants who did not have chronic diseases observed a significant decline (*P* < 0.001) in their average QoL score between baseline and follow-up assessment. In contrast, the average score in the psychological domain increased among participants with all chronic diseases except for cancer at the follow-up. For the other two fields, the average QoL scores improved significantly (*P* < 0.001) for almost all chronic diseases, irrespective of the presence or absence of the disease. However, the different domain scores were significantly lower (*P* < 0.05) among those with chronic conditions than those without at baseline and follow-up (Additional file 2: Table S1).

### Determinants of quality of life among different groups adjusted for dynamic changes

In the generalized estimating equation approach, after adjusting for other factors, we found that the age groups ≥ 26 years, females, hospital admission during COVID-19 illness, and chronic diseases were significantly associated with a lower physical (*P* < 0.001) and psychological QoL (*P* < 0.001) score (Table 3). Higher education and income also positively improved QoL scores in the social and environmental domains. In contrast, three or more comorbidities degraded the participants’ QoL in those domains (*P* < 0.001). After adjusting for all factors and their intra-group variations, a significant reduction in physical domain scores (*ß* = − 1.5, *P* < 0.001) and a significant increase in other domain scores were noted in follow-up interviews compared with baseline scores (*ß* = 1.8, *P* < 0.001; *ß* = 3.6, *P* < 0.001; *ß* = 3.2, *P* < 0.001).

**Table 3:** Factors associated with quality of life scores after adjusting for intra-individual changes between two interviews and for other factors by generalized estimating equation model

Variable	Categories	Difference in physical score		Difference in psychological score		Difference in social score		Difference in environmental score	
		Coefficient	*P*-*value*	Coefficient	*P*-*value*	Coefficient	*P*-*value*	Coefficient	*P*-*value*
Age	< 26 (Ref)	1		1		1		1	
	26–35	− 2.2	0.01	− 2.1	0.01	− 0.3	0.69	− 0.8	0.27
	36–45	− 3.4	< 0.001	− 3.9	< 0.001	− 0.1	0.86	− 0.8	0.28
	≥ 46	− 5.5	< 0.001	− 3.7	< 0.001	− 1.1	0.23	− 1.0	0.22
Gender	Male (Ref)	1		1		1		1	
	Female	− 2.2	< 0.001	− 3.1	< 0.001	− 0.4	0.46	0.2	0.57
Residence	Rural (Ref)	1		1		1		1	
	Urban/semi urban	− 0.7	0.31	− 0.1	0.87	0.5	0.41	0.9	0.09
Education	No/primary education (Ref)	1		1		1		1	
	Up to HSC	− 1.4	0.09	1.4	0.07	1.6	0.06	0.7	0.35
	Graduation/above	− 0.8	0.32	2.3	0.01	2.1	0.01	2.2	0.002
Employment status	Unemployed (Ref)	1		1		1		1	
	Employed	2.7	0.03	4.2	< 0.001	− 0.0	0.99	0.9	0.31
Income	< 20 000 (Ref)	1		1		1		1	
	20 001–40 000	1.7	0.002	2.5	< 0.001	2.5	< 0.001	0.8	0.09
	40 001–60 000	2.2	< 0.001	3.3	< 0.001	2.9	< 0.001	3.3	< 0.001
	> 60 000	2.3	0.001	2.8	< 0.001	6.8	< 0.001	4.8	< 0.001
Marital status	Unmarried (Ref)	1		1		1		1	
	Married	0.2	0.74	0.1	0.84	29.4	< 0.001	− 0.8	0.18
HCW	No (Ref)	1		1		1		1	
	Yes	1.0	0.08	1.0	0.11	0.7	0.27	− 0.2	0.71
Hospital admission	No (Ref)	1		1		1		1	
	Yes	− 2.9	< 0.001	− 2.7	< 0.001	− 2.9	< 0.001	0.2	0.69
Smoking status	Not smoker (Ref)	1		1		1		1	
	Smoker	− 0.2	0.74	− 1.4	0.01	− 1.7	0.002	− 0.1	0.81
	Past smoker	0.7	0.36	1.4	0.08	− 0.6	0.37	0.8	0.19
Number of chronic diseases	0 (Ref)	1		1		1		1	
	1	− 4.0	< 0.001	− 3.0	< 0.001	− 0.9	0.07	− 0.3	0.56
	2	− 6.9	< 0.001	− 5.9	< 0.001	− 3.6	< 0.001	− 1.1	0.11
	≥ 3	− 9.5	< 0.001	− 9.3	< 0.001	− 5.9	< 0.001	− 2.9	< 0.001
Follow-up	First (Ref)	1		1		1		1	
	Second	− 1.5	< 0.001	1.8	< 0.001	3.6	< 0.001	3.2	< 0.001

### Determinants of decline in quality of life of participants

On multivariable logistic regression analysis (Table 4), we observed that increasing age was significantly associated decline (26–35 years: a*OR* = 1.5, 95% *CI* 1.0–2.2; 36–45 years: a*OR* = 1.9, 95% *CI* 1.2–2.9; ≥ 46 years: a*OR* = 2.1, 95% *CI* 1.4–3.3) in social domain QoL, and females were 1.30 times more likely (a*OR* = 1.3, 95% *CI* 1.0–1.6) to have deteriorated social QoL than males during follow-up. Participants living in the urban or semi-urban areas were 49% less likely (a*OR* = 0.5, 95% *CI* 0.38–0.7) and 32% less likely (a*OR* = 0.7, 95% *CI* 0.5–0.9) to have a declined QoL in the psychological domain and the social domain, respectively, than rural people. Those who earned more than 60 000 BDT/month witnessed 1.5, 1.9, 2.2, and 1.5 times lower QoL than those with an income of less than 20 000 BDT in physical (a*OR* = 1.5, 95% *CI* 1.1–2.0), psychological (a*OR* = 1.9, 95% *CI* 1.4–2.5), social (a*OR* = 2.2, 95% *CI* 1.6–2.9), and environmental domains (a*OR* = 1.5, 95% *CI* 1.1–2.0), respectively. Participants admitted to hospitals during their COVID-19 infection showed a 1.32 times higher chance of a decreased environmental QoL score than those who did not (a*OR* = 1.3, 95% *CI* 1.1–1.6). People with three or more chronic diseases were 46% (a*OR* = 0.5, 95% *CI* 0.4–0.8) and 42% (a*OR* = 0.6, 95% *CI* 0.4–0.9) less likely to have a decreased QoL score in physical and psychological domains, respectively, than those without chronic diseases. The incidence of chronic diseases was associated with a 1.4 times higher chance of having a reduced physical domain score between two interviews (a*OR* = 1.4, 95% *CI* 1.0–1.8). Lastly, participants with a history of COVID-19 reinfection had 1.5 times and 1.7 times higher chance of having reduced QoL scores in psychological (a*OR* = 1.5, 95% *CI* 1.1–2.1) and social (a*OR* = 1.7, 95% *CI* 1.2–2.4) domains, respectively.

**Table 4:** Logistic regression model to identify factors that are associated decline in Quality of Life score from baseline to follow-up interview

Variable	Categories	Physical		Psychological		Social		Environmental	
		a*OR*	95% *CI*	a*OR*	95% *CI*	a*OR*	95% *CI*	a*OR*	95% *CI*
Age	< 26 (Ref)	1		1		1		1	
	26–35	1.1	0.8–1.5	1.1	0.8–1.5	1.5	1.0–2.2	0.9	0.7–1.4
	36–45	1.4	0.9–1.9	1.3	0.9–1.9	1.9	1.2–2.9	0.9	0.7–1.4
	≥ 46	1.4	0.9–2.1	1.4	0.9–2.0	2.1	1.4–3.3	0.8	0.5–1.2
Gender	Male (Ref)	1		1		1		1	
	Female	1.1	0.8–1.3	1.1	0.9–1.4	1.3	1.0–1.6	1.2	0.9–1.5
Residence	Rural (Ref)	1		1		1		1	
	Urban/semi urban	0.8	0.7–1.1	0.5	0.4–0.7	0.7	0.5–0.9	0.8	0.6–1.1
Educational status	No or primary education (Ref)	1		1		1		1	
	Up to HSC	1.4	1.0–1.9	0.8	0.6–1.1	0.7	0.5–1.0	0.9	0.7–1.3
	Graduate/above	1.2	0.9–1.7	0.7	0.5–1.0	0.8	0.6–1.2	0.9	0.6–1.3
Employment status	Unemployed (Ref)	1		1		1		1	
	Employed	1.1	0.7–1.7	1.1	0.6–1.8	0.6	0.4–1.0	0.9	0.6–1.6
Monthly family income in BDT	< 20 000 (Ref)	1		1		1		1	
	20 000–40 000	0.9	0.7–1.1	1.3	0.9–1.6	1.2	0.9–1.5	0.8	0.7–1.0
	40 001–60 000	1.1	0.8–1.4	1.4	1.1–1.9	1.4	1.0–1.9	1.1	0.8–1.5
	> 60 000	1.5	1.1–2.0	1.9	1.4–2.5	2.2	1.6–2.9	1.5	1.1–2.0
Marital status	Single (Ref)	1		1		1		1	
	Married	1.2	0.9–1.5	1.0	0.8–1.4	1.8	1.3–2.4	1.2	0.9–1.6
HCW	No (Ref)	1		1		1		1	
	Yes	0.7	0.6–0.9	0.5	0.4–0.7	0.8	0.6–1.0	0.8	0.6–1.0
Hospital admission	No (Ref)	1		1		1		1	
	Yes	1.2	0.9–1.4	0.9	0.8–1.2	0.8	0.7–1.0	1.3	1.1–1.6
Smoking status	Not smoker (Ref)	1		1		1		1	
	Smoker	0.9	0.7–1.2	0.9	0.8–1.2	0.9	0.7–1.2	1.1	0.8–1.4
	Past smoker	0.9	0.7–1.2	1.4	1.0–1.9	0.9	0.7–1.3	1.2	0.8–1.6
Number of chronic diseases	0 (Ref)	1		1		1		1	
	1	0.7	0.5–0.8	0.8	0.6–0.9	0.9	0.7–1.1	1.0	0.8–1.3
	2	0.9	0.7–1.3	0.7	0.5–1.1	1.1	0.7–1.5	0.9	0.7–1.4
	≥ 3	0.5	0.4–0.8	0.6	0.4–0.9	0.7	0.4–1.0	0.9	0.7–1.4
New chronic disease	No (Ref)	1		1		1		1	
	Yes	1.4	1.0–1.8	1.1	0.9–1.5	1.1	0.9–1.5	1.1	0.8–1.5
COVID-19 vaccination	Yes (Ref)	1		1		1		1	
	No	0.9	0.8–1.1	1.1	0.9–1.4	1.2	0.9–1.5	1.1	0.91.3
COVID-19 re-infection	No (Ref)	1		1		1		1	
	Yes	0.9	0.6–1.2	1.5	1.1–2.1	1.7	1.2–2.4	0.8	0.5–1.1

## Discussion

Before the widespread global vaccination, the COVID-19 pandemic was responsible for the deaths of millions and had devastating economic consequences. The aftermath of the pandemic might continue to affect people directly through its long-term physical and psychological sequels and indirectly through its negative socio-economic impacts [19, 20]. In this study, we focused on the long-term effects of COVID-19. The COVID-19 recovered patients were surveyed twice-approximately, 6 months (baseline) and 18 months (follow-up) after recovery.

There was, on average, a statistically significant decline in the physical domain score and a substantial increase in the participants’ psychological, social, and environmental domain scores from baseline to follow-up interview. However, this varied across different participants’ characteristics. Taking the intra-individual variations between the two interviews into account, we found that higher age, female sex, history of hospital admission during COVID-19, smoking, and a higher number of chronic diseases were associated with a lower score in different domains. On the other hand, higher education, employment, and marriage were associated with higher scores in various domains. This is similar to previous studies on QoL of COVID-19 patients conducted during their active illness or between 1 and 6 months after recovery, where older age, female sex, hospitalization history, unemployed, and comorbidities were reported to be associated with low levels of QoL [21]. Contrary to a study in Pakistan [22], which showed an improvement in physical QoL over 6 months after diagnosis, we found an overall decrease in physical QoL over an extended period. In the multivariable logistic regression analysis, we found several independent determinants of this decline, including the new onset of chronic disease and reinfection with SARS-CoV-2. Although the overall QoL score in the physical domain increased and in other domains decreased, each patient either experienced an increase or decrease or no change in QoL scores between the first and second interviews. Therefore, we sorted out the participants who experienced a decline in the score and explored the determinants of the decrease through multivariable regression.

The multivariate logistic regression analysis also revealed that after adjusting for other variables, the decline in the physical domain occurred mainly in participants from the highest income category (> 60 000 BDT) and participants other than HCWs. Interestingly, the reduction was significant in those who did not have comorbidities during the first interview but who later developed chronic disease. This indicates that the average decline in the physical domain scores in the adult groups (36–45 and ≥ 46 years), as found in the bivariate analysis, was because of the new onset of chronic disease within one and a half years after COVID-19 infection. On the other hand, people from higher socio-economic categories were more likely to have insufficient physical activity [23] even after quarantine and movement restrictions had been lifted, and this may explain their propensity to develop chronic disease and suffer physical decline. Nonetheless, as participants had 1 year increase in age between the two interview periods, aging could be a determinant of a negative trend in physical QoL. One study conducted among older people in Bangladesh found a lower average QoL score [24], even lower than ours. HCWs are likely to be more cautious about their health because of their high-risk perception and knowledge about COVID-19 [15, 25, 26], which might have allowed them to maintain good health over time.

Although the average psychological domain score improved in all patients, participants who suffered reinfection by SARS-CoV-2 between the first and second interviews were significantly more likely to decline the score. Moreover, the odds of the decline were higher in those residing in rural areas and having a higher income (> 40 000 BDT). COVID-19 can lead to a general deterioration of the affected person’s mental health [27–29]. In addition, the rural economy of Bangladesh suffered a severe adverse impact of the COVID-19 pandemic [30]. Making up for that loss subsequently would have cast immense psychological stress on those who recovered from COVID-19 in rural compared to urban areas. Participants from high-income categories might have had fallen into difficult social and economic circumstances which is deeply interconnected with a person’s psychological health [28]. This study also found that the highest monthly income category (> 60 000 BDT) was significantly associated with a decline in social and environmental domains. The odds of decline in psychological domain score were lower in participants having chronic diseases at the first interview probably because a proportion of those without chronic disease at baseline developed the chronic disease at follow-up increasing the effect size in the second group.

The WHOQOL-BREF instrument measures social domain scores based on participants’ perceptions of their relationships, sex life, and support from friends. According to our findings, predominantly older adults and females were affected by this shift in perspective. After multivariable adjustments, other domains remained unaffected by the participant’s age or sex. Besides, rural residence, a higher monthly income (40 001–60 000, and > 60 000 BDT), being married, and reinfection of COVID-19 between the first and second interviews was independently associated with a decline in the social domain. This finding is in line with previous evidence, as female sex and older age were reported to be associated with low QoL in many studies conducted on the mental health impact of COVID-19 [21]. We found the same picture in our bivariate and GEE analysis. COVID-19 sufferers were reported to experience a more significant effect on family activity and sex life [21]. Additionally, reinfection and new-onset chronic disease might have created increased needs for social support, only to remain unmet by the equally affected community. Sexual satisfaction could be the primary modifier of social QoL for married individuals. However, despite being more socially interconnected, rural residents might have failed to avail themselves of the expected levels of support because of the higher economic impact of COVID-19 in the rural areas [30].

In the environmental domain, besides higher monthly income, another independent predictor of the score was the history of hospital admission due to COVID-19, an indicator of severe disease. Health and social care accessibility and availability, which are essential components of the environmental domain [17], might have been inadequate in these participants needing follow-up hospital visits for recovery and/or maintenance of bodily functions and mental health. Several previous studies [31–33] conducted on patients admitted to the hospital due to severe disease and critical illness reported that these patients suffered a low QoL for as long as 6 months after discharge from the hospital, particularly in the physical and psychological components.

Our analysis revealed that the new onset of chronic diseases after recovery from COVID-19 was a significant negative determinant of QoL among the sufferers. A recent study exploring the QoL among type 2 DM patients found a very low average score in all four domains, which supports our assumption [34]. On the other hand, as chronic disease might be co-incident with older age [35], the lower QoL score could be due to aging [24].

Regrettably, our study could not compare the QoL scores between individuals who have not been infected with COVID-19 and those who have recovered from the disease. Nevertheless, earlier investigations conducted among a healthy population in Bangladesh indicate that adolescents and adults had an average QoL score of 80–90 between 2005 and 2007 [36, 37]. In contrast, our study found lower mean QoL scores among participants. While one would expect a general improvement in QoL after recovering from COVID-19, the contrasting scores observed in our study may reflect the pandemic’s adverse socio-economic effects on the country’s population. However, drawing realistic conclusions on this situation is challenging due to the absence of an actual control group.

Our study highlighted the fact that COVID-19 pandemic and the drastic control measures taken during that period, had long time consequences among the persons affected by the disease. Although many individuals had been adapting well with time, a considerable number experienced a decline in their quality of life. Nonetheless, authorities and policy makers could take the determinants of decline in consideration and plan necessary actions to reverse the process of decline. Especially, risk of re-infection could be a major mediator of decline in QoL among recovered victims of the disease. Lessons learnt from COVID could be applied for unforeseen emergence of diseases in the future. Rather than applying nonchalant or drastic measures, applying dynamic control mechanisms based on realistic unbiased calculations [38, 39] could be helpful to effectively curtail highly infectious diseases like COVID-19 ensuring that all state systems are running without being tipped off.

This study had some limitations. First, many participants were lost from follow-up. Second, an evaluation of the effect of socio-cultural determinants like health service availability, economic security, rehabilitative measures, and health-seeking behavior on the QoL could not be done. Third, the impacts of persistent and debilitating symptoms after COVID-19 were not explored. Fourth, there were no true controls to compare the QoL scores with that of individuals who did not suffer from COVID-19. However, our study was one of the few which reported the QoL of COVID-19 after an extended duration and described possible implications for policy-level strategies to prevent further demise and rehabilitate these individuals to total health.

## Conclusions

The present study found that the QoL of COVID-19 recovered people improved over more than 1 year after recovery, particularly in psychological, social, and environmental domains. However, age, sex, the severity of COVID-19, smoking habits, and comorbidities were significantly associated with reduced QoL. Events of reinfection and the emergence of chronic disease were independent determinants of the decline in QoL scores in psychological, social, and physical domains, respectively. Based on our study findings, we have the following recommendations: (1) COVID-19-recovered people should be monitored for early diagnosis, prompt management of chronic diseases, and encouragement of necessary preventive measures to reduce the risk for further illness. (2) Adults, women, and people who recovered from severe COVID-19 should be given special attention regarding strategies taken for recovery to total health. (3) Psychological and social support should be encouraged for people who become re-infected with COVID-19, (4) action research should be conducted into QoL in Bangladesh and on the impacts of COVID-19 over time, and (5) strong policies should be adopted to discourage and reduce or stop smoking in the country.

### Supplementary Information

**Additional file 1: Figure S1.** Flow chart of participant selection and data collection. **Figure S2.** Pattern of change in score in physical, psychological, social and environmental domains of quality of life. **Figure S3.** Pattern of changes in overall quality of life and health satisfaction over the period. **Figure S4.** Onset of new chronic disease and percentage of re-infection among the recovered COVID-19 participants during second follow-up.

**Additional file 2: Table S1.** Comparison of quality of life between baseline and follow-up interviews in relation to presence or absence of individual chronic diseases.

## Data Availability

The data underlying the results presented in this study will be provided on reasonable request to Dr. Delwer H. Hawlader. Email: mohammad.hawlader@northsouth.edu.

## Abbreviations

ANOVA: Analysis of Variance
a*OR*: Adjusted odd ratio
BDT: Bangladesh Taka
*CI*: Confidence interval
CKD: Chronic kidney disease
GEE: Generalized estimating equation
HCW: Health care worker
HRQoL: Health related quality of life
QoL: Quality of Life
RT-PCR: Reverse transcription-polymerase chain reaction
SARS: Severe acute respiratory syndrome
SARS-CoV-2: Severe acute respiratory syndrome coronavirus 2
*SD*: Standard deviation
WHO: World Health Organization
WHOQOL-BREF: World Health Organization quality of life brief version
