# Supplementary material for: Quality of life of COVID-19 recovered patients: a 1-year follow-up study from Bangladesh
Source: Infect Dis Poverty. 2023 Aug 25;12:79. doi: 10.1186/s40249-023-01125-9 (PMC10463646; doi:10.1186/s40249-023-01125-9)
Supplement: Supplementary file 1 — Additional file 1: Figure S1. Flow chart of participant selection and data collection. Figure S2. Pattern of change in score in physical, psychological, social and environmental domains of quality of life. Figure S3. Pattern of changes in overall quality of life and health satisfaction over the period. Figure S4. Onset of new chronic disease and percentage of re-infection among the recovered COVID-19 participants during second follow-up. [file 40249_2023_1125_MOESM1_ESM.docx]

**Supplementary Figure 2: Pattern of change in score in physical, psychological, social and environmental domains of quality of life (■ Increased; ■ Unchanged; ■ Decreased)**


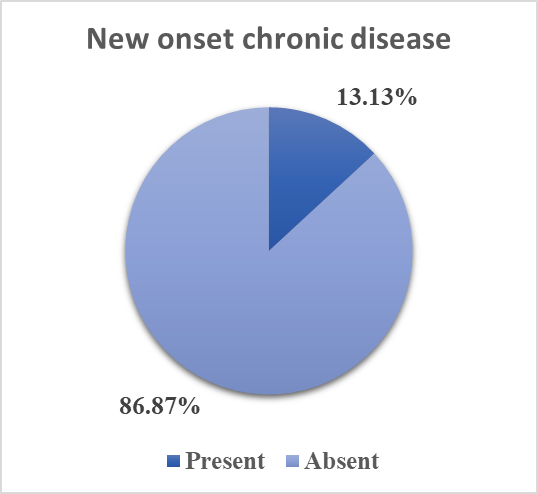

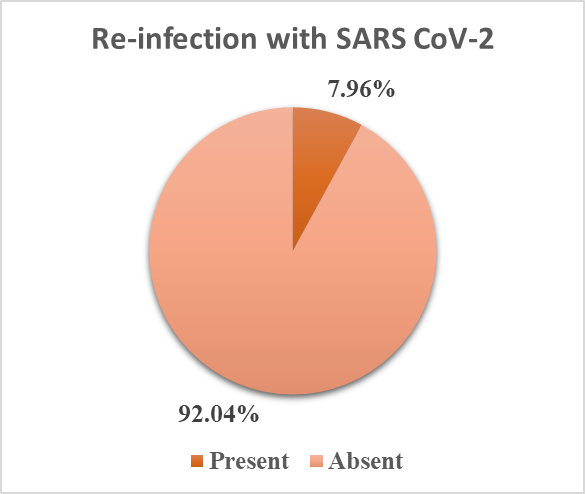


**Supplementary Figure 3: Onset of new chronic disease and percentage of re-infection among the recovered COVID-19 participants during second follow-up**
