# Supplementary material for: Quality of life of COVID-19 recovered patients: a 1-year follow-up study from Bangladesh
Source: Infect Dis Poverty. 2023 Aug 25;12:79. doi: 10.1186/s40249-023-01125-9 (PMC10463646; doi:10.1186/s40249-023-01125-9)
Supplement: Supplementary file 2 — Additional file 2: Table S1. Comparison of quality of life between baseline and follow-up interviews in relation to presence or absence of individual chronic diseases. [file 40249_2023_1125_MOESM2_ESM.docx]

**Table S1: Comparison of quality of life between baseline and follow-up interviews in relation to presence or absence of individual chronic diseases**

| **Variable** | | **Physical** | | | **Psychological** | | | **Social** | | | **Environmental** | | |
| --- | --- | --- | --- | --- | --- | --- | --- | --- | --- | --- | --- | --- | --- |
|  |  | **1st visit** | **2nd visit** | ***P*** | **1st visit** | **2nd visit** | ***P*** | **1st visit** | **2nd visit** | ***P*** | **1st visit** | **2nd visit** | ***P*** |
|  |  | **Mean (SD)** | **Mean (SD)** |  | **Mean (SD)** | **Mean (SD)** |  | **Mean (SD)** | **Mean (SD)** |  | **Mean (SD)** | **Mean (SD)** |  |
| **Hypertension** | No | 70.3  (14.0) | 67.6  (14.4) | <0.001 | 65.5(14.9) | 65.7  (14.0) | 0.56 | 61.3  (19.7) | 65.5  (17.8) | <0.001 | 63.0  (12.6) | 66.0  (11.4) | <0.001 |
|  | Yes | 63.9  (13.8) | 62.3  (14.4) | 0.05 | 58.8(16.2) | 62.5  (13.8) | <0.001 | 62.4  (17.8) | 66.8  (16.0) | <0.001 | 62.5  (13.5) | 65.4  (11.9) | <0.001 |
|  | *P-value* | <0.001 | <0.001 |  | <0.001 | <0.001 |  | 0.29 | 0.17 |  | 0.46 | 0.32 |  |
| **Diabetes** | No | 70.6  (13.9) | 67.7  (14.5) | <0.001 | 65.5(15.0) | 65.9  (13.9) | 0.36 | 61.5  (19.7) | 66.0  (17.7) | <0.001 | 63.0  (12.7) | 66.1  (11.5) | <0.001 |
|  | Yes | 61.8  (13.4) | 61.1  (13.3) | 0.45 | 57.8(15.3) | 61.2  (13.8) | <0.001 | 61.8  (17.7) | 64.3  (16.1) | 0.02 | 62.6  (13.4) | 64.6  (11.5) | 0.02 |
|  | *P-value* | <0.001 | <0.001 |  | <0.001 | <0.001 |  | 0.78 | 0.07 |  | 0.56 | 0.02 |  |
| **Heart disease** | No | 69.9  (14.1) | 67.0  (14.6) | <0.001 | 64.9(15.2) | 65.4  (14.1) | 0.17 | 61.8  (19.5) | 65.9  (17.6) | <0.001 | 63.1  (12.7) | 66.1  (11.5) | <0.001 |
|  | Yes | 59.5  (12.2) | 62.0  (13.2) | 0.04 | 56.0  (15.3) | 61.3  (13.0) | <0.001 | 57.5  (17.5) | 63.3  (15.9) | <0.001 | 61.1  (14.0) | 63.4  (10.9) | 0.08 |
|  | *P-value* | <0.001 | <0.001 |  | <0.001 | <0.001 |  | 0.005 | 0.06 |  | 0.05 | 0.003 |  |
| **Asthma/ COPD** | No | 70.3  (14.0) | 67.4  (14.4) | <0.001 | 65.3  (15.0) | 65.5  (13.9) | 0.59 | 62.2  (19.3) | 65.9  (17.4) | <0.001 | 63.2  (12.7) | 66.1  (11.4) | <0.001 |
|  | Yes | 60.4  (12.4) | 60.6  (14.2) | 0.84 | 55.4(15.2) | 61.6  (14.8) | <0.001 | 56.1  (19.0) | 64.7  (17.9) | <0.001 | 60.8  (13.8) | 63.9  (12.3) | 0.01 |
|  | *P-value* | <0.001 | <0.001 |  | <0.001 | <0.001 |  | <0.001 | 0.28 |  | 0.004 | 0.003 |  |
| **CKD** | No | 69.5  (14.2) | 66.9  (14.5) | <0.001 | 64.6  (15.2) | 65.2  (14.0) | 0.06 | 61.7  (19.4) | 65.9 (17.5) | <0.001 | 63.1  (12.8) | 65.8  (11.4) | <0.001 |
|  | Yes | 60.0  (10.6) | 59.7  (13.7) | 0.87 | 55.1  (17.1) | 61.2  (13.8) | 0.02 | 55.8  (18.3) | 61.6  (17.0) | 0.03 | 57.4  (11.9) | 67.4  (14.5) | <0.001 |
|  | *P-value* | <0.001 | <0.001 |  | <0.001 | 0.01 |  | 0.01 | 0.03 |  | 0.001 | 0.23 |  |
| **Cancer** | No | 69.1  (14.1) | 66.8  (14.6) | <0.001 | 64.3  (15.0) | 65.5  (14.1) | 0.001 | 61.8  (19.6) | 65.9  (17.6) | <0.001 | 62.8  (12.7) | 66.2  (11.6) | <0.001 |
|  | Yes | 59.3  (8.3) | 58.4  (14.3) | 0.60 | 51.9  (14.0) | 55.9  (15.3) | 0.08 | 49.9  (17.9) | 58.2  (18.1) | 0.002 | 62.4  (17.8) | 63.6  (12.6) | 0.67 |
|  | *P-value* | <0.0001 | <0.001 |  | <0.001 | <0.001 |  | <0.001 | 0.001 |  | 0.78 | 0.05 |  |
